# The Potential Complementary Role of Using Chinese Herbal Medicine with Western Medicine in Treating COVID-19 Patients: Pharmacology Network Analysis

**DOI:** 10.3390/ph15070794

**Published:** 2022-06-26

**Authors:** Yi-Chin Lu, Liang-Wei Tseng, Yu-Chieh Huang, Ching-Wei Yang, Yu-Chun Chen, Hsing-Yu Chen

**Affiliations:** 1 Division of Chinese Internal Medicine, Center for Traditional Chinese Medicine, Chang Gung Memorial Hospital, Taoyuan 33378, Taiwan; yichinlu.lu@gmail.com (Y.-C.L.); frank.tseng.sr@gmail.com (L.-W.T.); 8905002@adm.cgmh.org.tw (C.-W.Y.); 2 Department of Medical Imaging and Intervention, Chang Gung Memorial Hospital, Keelung 20401, Taiwan; 8702022@chmg.org; 3 School of Traditional Chinese Medicine, College of Medicine, Chang Gung University, Taoyuan 33302, Taiwan; 4 Faculty of Medicine, School of Medicine, National Yang-Ming Chiao Tung University, Taipei 11221, Taiwan; ycchen22@vghtpe.gov.tw; 5 Institute of Hospital and Health Care Administration, National Yang-Ming Chiao Tung University, Taipei 11221, Taiwan; 6 Department of Family Medicine, Taipei Veterans General Hospital, Taipei 11217, Taiwan; 7 Graduate Institute of Clinical Medical Sciences, College of Medicine, Chang Gung University, Taoyuan 33302, Taiwan

**Keywords:** anti-inflammation, Chinese herbal medicine, coronavirus disease 2019 (COVID-19), severe acute respiratory syndrome coronavirus 2 (SARS-CoV-2), pharmacology network analysis, Western medicine

## Abstract

The severe acute respiratory syndrome coronavirus 2 (SARS-CoV-2) caused a global pandemic in 2019—coronavirus disease (COVID-19). More and more Western medicine (WM) and Chinese herbal medicine (CHM) treatments have been used to treat COVID-19 patients, especially among Asian populations. However, the interactions between WM and CHM have not been studied. This study aims at using the network pharmacology approach to explore the potential complementary effects among commonly used CHM and WM in a clinical setting from a biomolecular perspective. Three well-published and widely used CHM formulas (National Research Institute of Chinese Medicine 101 (NRICM101), Qing-Fei-Pai-Du-Tang (QFPDT), Hua-Shi-Bai-Du-Formula (HSBDF)) and six categories of WM (Dexamethasone, Janus kinase inhibitors (JAKi), Anti-Interleukin-6 (Anti-IL6), anticoagulants, non-vitamin K antagonist oral anticoagulants (NOAC), and Aspirin) were included in the network pharmacology analysis. The target proteins on which these CHM and WM had direct effects were acquired from the STITCH database, and the potential molecular pathways were found in the REACTOME database. The COVID-19-related target proteins were obtained from the TTD database. For the three CHM formulas, QFPDT covered the most proteins (714), and 27 of them were COVID-19-related, while HSBDF and NRICM101 covered 624 (24 COVID-19-related) and 568 (25 COVID-19-related) proteins, respectively. On the other hand, WM covered COVID-19-related proteins more precisely and seemed different from CHM. The network pharmacology showed CHM formulas affected several inflammation-related proteins for COVID-19, including IL-10, TNF-α, IL-6, TLR3, and IL-8, in which Dexamethasone and Aspirin covered only IL-10 and TNF-α. JAK and IL-6 receptors were only inhibited by WM. The molecular pathways covered by CHM and WM also seemed mutually exclusive. WM had advantages in cytokine signaling, while CHM had an add-on effect on innate and adaptive immunity, including neutrophil regulation. WM and CHM could be used together to strengthen the anti-inflammation effects for COVID-19 from different pathways, and the combination of WM and CHM may achieve more promising results. These findings warrant further clinical studies about CHM and WM use for COVID-19 and other diseases.

## 1. Introduction

The severe acute respiratory syndrome coronavirus 2 (SARS-CoV-2) has imposed a heavy burden on the healthcare system and caused the pandemic of coronavirus disease 2019 (COVID-19) in 2019. Many therapeutic agents have been used to treat the disease according to the British Medical Journal (BMJ) living review ([1], until 6 APR 2021) and WHO guidelines (until 24 SEP 2021). For example, corticosteroids are likely to reduce mortality, lower the need for mechanical ventilation, and increase the number of ventilator-free days. Anti-IL6 lessens the need for mechanical ventilation and the duration of hospitalization. Janus kinase inhibitors (JAKi) probably reduce the need and the duration of mechanical ventilation. In Asian countries, several Chinese herbal medicine (CHM) treatments have been used with Western medicine (WM) to treat COVID-19 patients [2]. Reviews of CHM on COVID-19 and their possible therapeutic mechanisms have been widely researched [3,4,5,6,7,8,9,10,11,12,13]. Sun X et al. concluded that commonly used CHM achieves its therapeutic effect by modulating the target proteins, particularly by anti-inflammatory, anti-viral, and immune regulation [11].

However, CHM is seldom used alone in clinical practice when dealing with life-threatening and pandemic diseases such as COVID-19. Thus, the interactions between WM and CHM, especially those prescribed to treat COVID-19, should be an essential issue and have not been comprehensively studied. One review indicated that combined treatment of COVID-19 with CHM and WM may have an add-on effect in symptom relief and preventing disease progression [2]. However, the quality and evidence of the research were insufficient, the WM mentioned in this study was not totally compatible with the standard treatment guideline, and the mechanism of how CHM and WM achieve synergistic effects was lacking.

Several CHM formulas based on traditional Chinese medicine (TCM) theory were introduced in treatment guidelines published by authorities and showed evidence for their efficacy toward COVID-19 [14,15]. COVID-19 is recognized a kind of plague, namely “wen yi” in Chinese, in TCM theory, which presented pathogens with epidemic or pandemic spread. For plague management, individualized treatments aiming at the interactions between pathogens and human bodies were developed by TCM doctors. However, based on the different geolocations, climate and constitutions of populations, different CHM prescriptions may be made for one pathogen. The National Research Institute of Chinese Medicine 101 (NRICM101), named Taiwan Chingguan Yihau, was developed by summarizing the clinical courses of COVID-19 and the prior experience of the 2003 SARS outbreak in Taiwan and tended to dispel pathogens [16,17]. Qing-Fei-Pai-Du-Tang (QFPDT) is a modified combination of several ancient formulas which were already widely used for over 2000 years in the treatment of various kinds of virus infection [18]. Hua-Shi-Bai-Du-Formula (HSBDF) was formulated based on another theory of TCM, in which “cold” and “humidity” were considered as the primary pathogenesis of COVID-19 [13].

Among them, NRICM101 had benefits in the patients who were older, with more complications and showing no improvement after 21 days of hospitalization [15]. QFPDT showed a relative reduction in 50% of in-hospital COVID-19 related mortality compared to the control group among 8939 patients in an observation study [19]. In one study that included 55 severe patients, HSBDF exhibited a significantly higher ratio of nucleic acid negative conversion, shorter time of virus RNA clearance, more improvement of inflammation markers, and more lung lesion absorbed in CT image [20]. The clinical remission time of Group HSBDF plus anti-virus therapy was significantly shorter among 60 patients in a non-randomized controlled trial [21].

Briefly, NRICM101 can be used in mild cases, and the use of NRICM101 for moderate–severe illness patients is probable but still under investigation. HSBDF is recommended in severe cases. QFPDT is suitable for mild to severe patients, and it can be used in critically ill patients. The detailed indications and usage of NRICM101 is described in the Chinese Medicine Clinical Practice Guideline on COVID-19 in Taiwan [15], while the indications and usage of QFPDT and HSBDF is mentioned in the Diagnosis and Treatment Protocol for Novel Coronavirus Pneumonia (Trial Version 7) in China [14]. These three widely used CHM formulas, along with six categories of WM, were chosen to evaluate through the network pharmacology approach in our study.

Because of the high contagion and variability of novel epidemic diseases such as COVID-19, scientists face the challenge of exploring treatment candidates in currently existing drugs as comprehensively and expeditiously as possible based on how the pathogen invades the human body when precise therapy is not yet available. This strategy is slightly different from the previous new drug development model that required a long time and multiple phases. With the characteristics of having huge databases and clear theoretical sources of each pathway, network pharmacology is particularly suitable for quickly screening out potential drugs. This methodology has developed in the past few years [22] and has already been utilized during this pandemic, both in WM and CHM [23,24]. However, researchers have seldom focused on another advantage of network pharmacology, which is to evaluate the strengths and weaknesses of current treatment for a disease, especially when a combination of drugs are provided. A previous study on allergic rhinitis was conducted [25] when CHM and WM were applied together, but there is no such analysis for COVID-19.

This study aims to highlight important proteins and molecular pathways and speculate on the synergistic effects from the biomolecular perspectives of two kinds of medicine through illustrating the network pharmacology. The results of this study will enhance the understanding of the possible mechanism of the complementary effect between CHM and WM. This analytical method will provide a potential model due to its accessibility when confronting novel diseases in the future.

## 2. Results

### 2.1. Ingredients and Chemical Compounds of Commonly Prescribed CHM in COVID-19

A total of six groups of WM and three kinds of CHM with 35 herbal ingredients were listed in Table 1 (details and the botanical names of CHM are found in Supplementary in Material S1 and S2). A total of 14 herbs are in the HSBDF, 10 herbs in the NRICM101, and 21 herbs in the QFPDT. Among them, QFPDT had the most chemical compounds (*n* = 1430), followed by HSBDF (*n* = 1036) and NRICM101 (*n* = 838). A detailed list of chemical compounds is provided in Supplementary Material S3.

### 2.2. Network Pharmacology of CHM and WM

**Figure 1:**
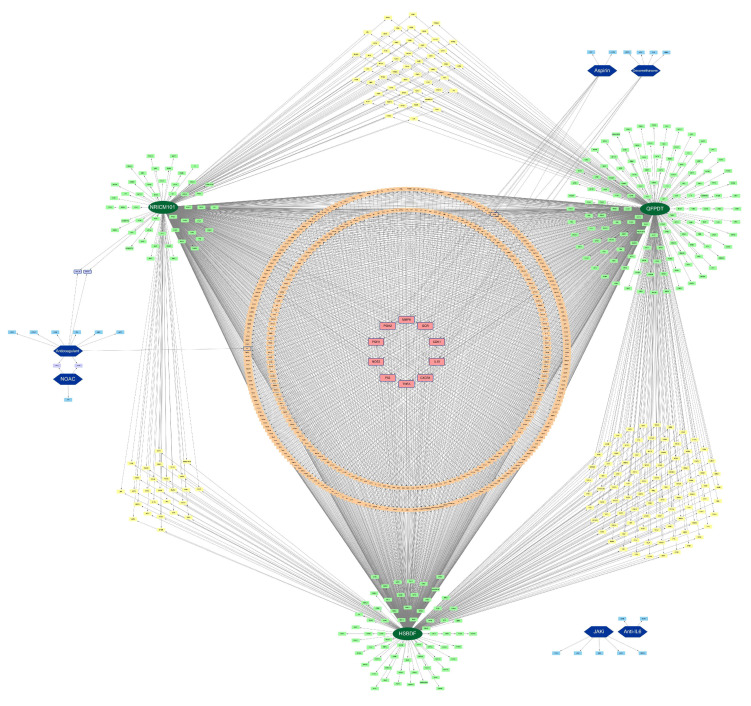
Target–drug interaction network of the 3 CHM formulas and 6 WM categories.

Figure 1 revealed all the binding proteins of the WM and CHM. NRICM101 affected 568 proteins, while QFPDT connected to 714 proteins and HSBDF linked to 624 proteins (for details, see Supplementary Material S4). The proteins covered by CHM formulas are mostly overlapping (848 proteins are covered by CHM, of which 599 are covered by at least two CHM). CHM showed a more extensive connection with proteins while WM bonded with proteins more specifically. Some proteins, including CDK1, GCR, MMP9, PGH1, PGH2, NOS3, P53, TNF-α, CXCR4, and IL-10, were covered by more CHM and WM simultaneously. Among them, CDK1, GCR, TNF-α, CXCR4, and IL-10 were involved with Dexamethasone; NOS3, P53, MMP9, PGH1, PGH2 and IL-10 were related to Aspirin. Aspirin and Dexamethasone seemed to have higher relevance with CHM, as seven out of nine Aspirin target proteins and one-half of Dexamethasone target proteins also connected with CHM. In contrast, Janus kinase inhibitor (JAKi) and Anti-Interleukin-6 (Anti-IL6) had no mutual target proteins with CHM and showed no significant interaction with all other drugs.

Dark green oval labels indicate CHM, and dark blue hexagonal labels indicate WM. Light green labels indicate proteins that are only affected by one CHM, and light blue labels indicate proteins that are only affected by one WM. The target proteins in the yellow labels are affected by any two of the three CHM. The orange labels are affected by three kinds of drugs, and pink labels are proteins that are affected by four different drugs. The target proteins with a blue margin are affected by WM. The short names of proteins are quoted from the STITCH database.

### 2.3. COVID-Related Target Protein Covered by CHM and WM

**Figure 2:**
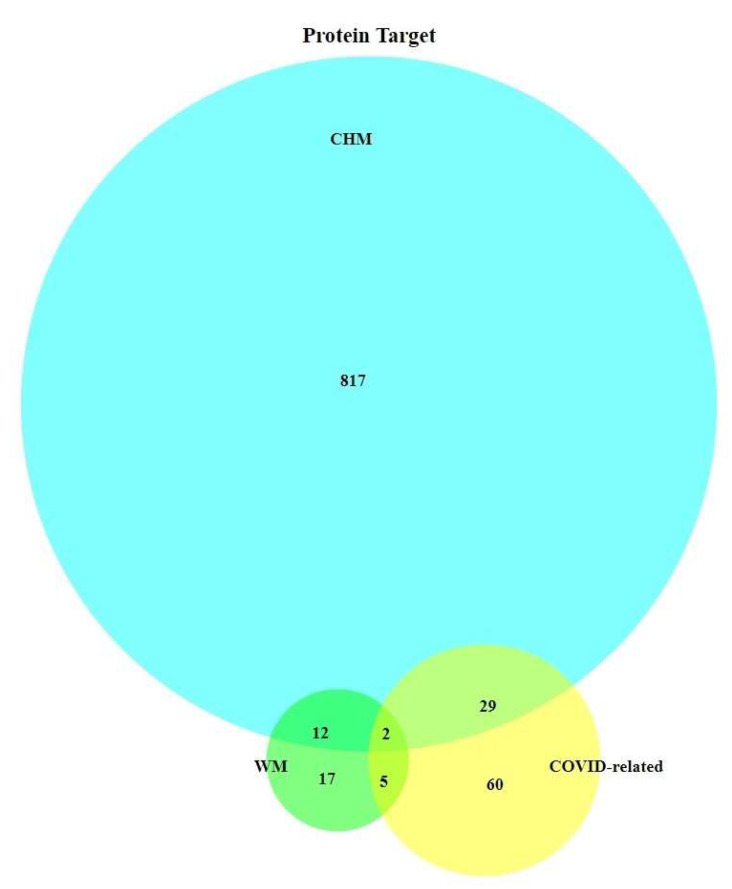
Venn diagram of protein targets.

**Figure 3:**
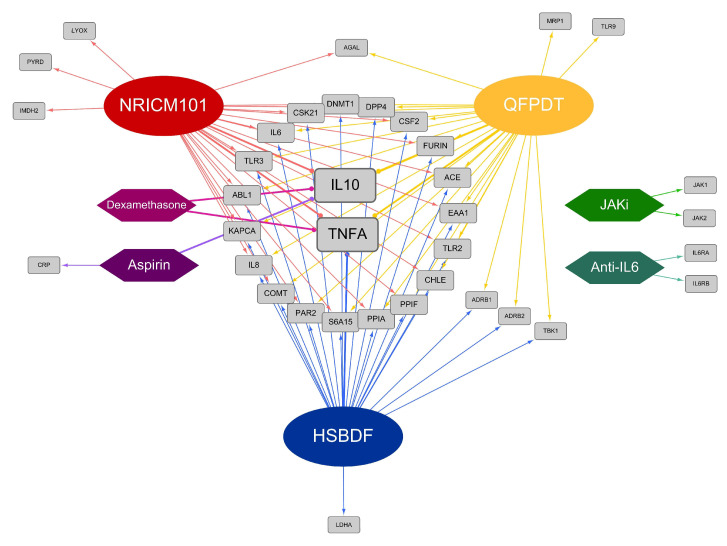
Target–drug interaction network of COVID-related protein in 3 CHM formulas and 6 WM categories.

We summarized a total of 96 target proteins that were associated with COVID-19, and more than one-third of them were found to be directly related to the chosen drugs, which is displayed in Figure 2. CHM affected more proteins, but only a relatively small proportion (31 out of 816) overlapped with COVID-related proteins. On the other hand, WM affected 40 proteins and 7 were related to COVID-19. Two specific proteins, TNF-α and IL-10 (depicted in Figure 3), were covered by both CHM and WM in COVID-related proteins.

**Figure 4:**
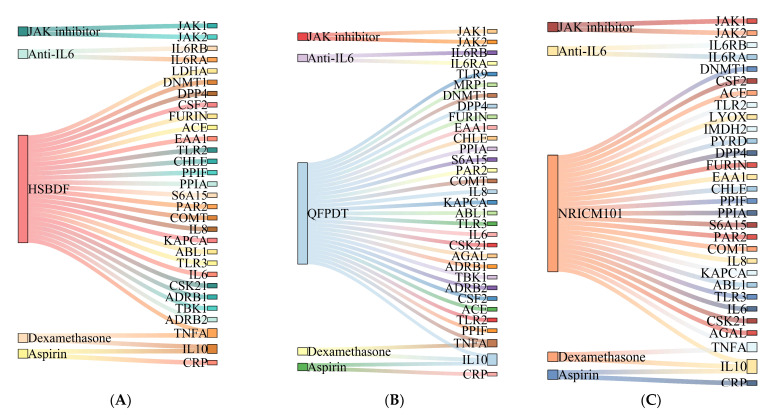
(**A**) Interaction between HSBDF plus WM and COVID-related proteins in a Sankey diagram. (**B**) Interaction between QFPDT plus WM and COVID-related proteins in a Sankey diagram. (**C**) Interaction between NRICM101 plus WM and COVID-related proteins in a Sankey diagram.

Network pharmacology demonstrated the relationships of COVID-related proteins affected by CHM and WM (Figure 3). QFPDT, NRICM101, and HSBDF shared most target proteins, but some particular proteins can only be influenced by certain CHM. Among them, MRP1 and TLR9 were connected to QFPDT; LYOX, PYRD, and IMDH2 were linked to NRICM101, while LDHA was only affected by HSBDF. The target proteins of each CHM are presented in Figure 4. Figure 3 also shows that CHM formulas affected on several inflammation-related proteins for COVID-19, e.g., IL-10, TNF-α, IL-6, TLR3, and IL-8. IL-10 was covered by Aspirin and Dexamethasone, and TNF-α were covered by Dexamethasone. In addition, JAKi and IL-6 receptors were only inhibited by WM.

### 2.4. Interaction and Relationships between CHM and WM

**Figure 5:**
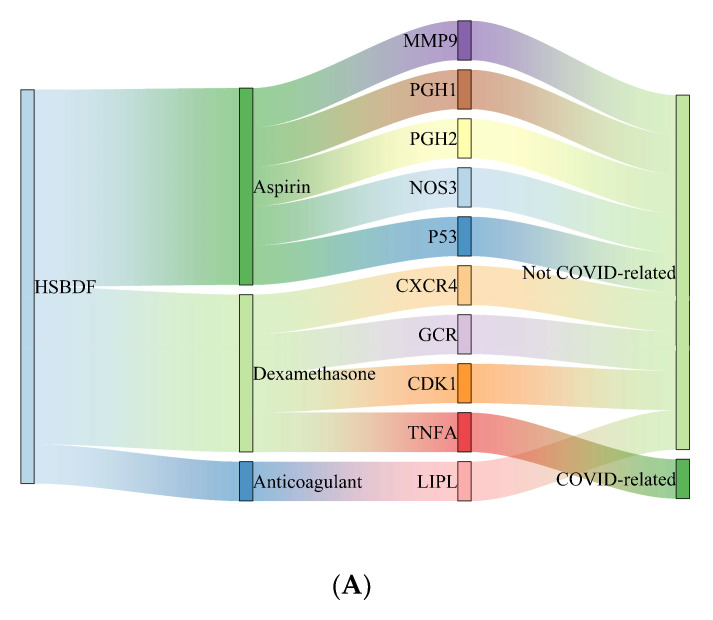

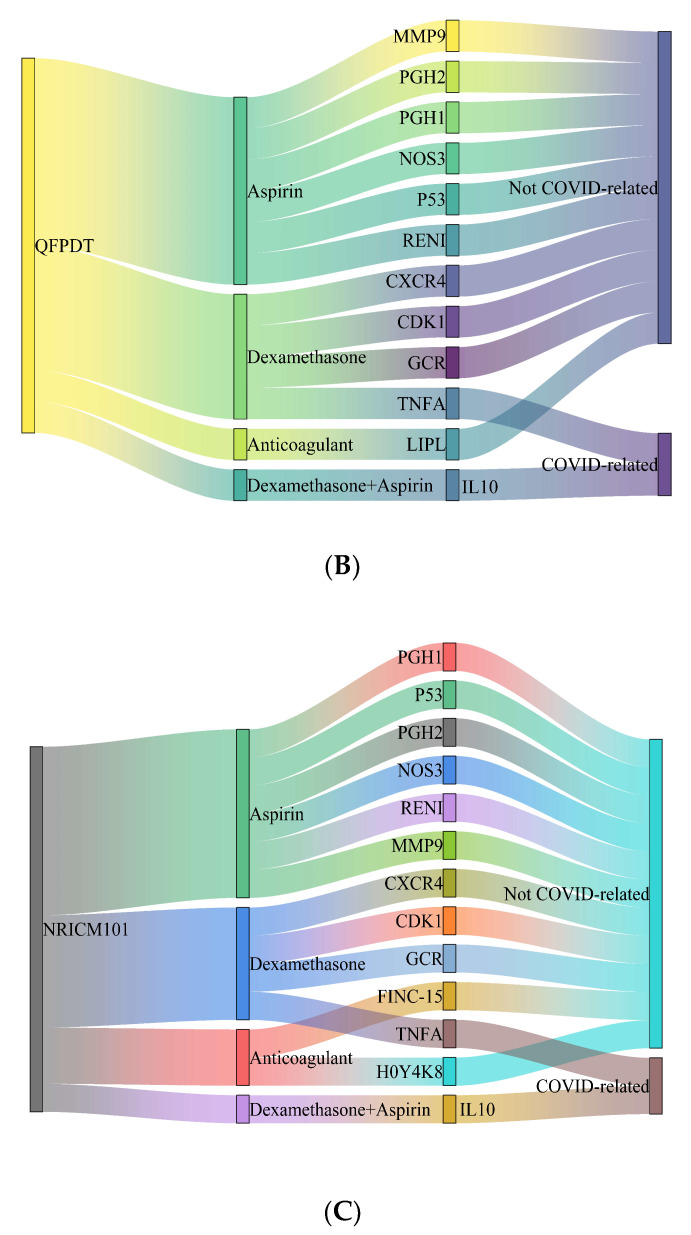
(**A**)Target–drug interaction when HSBDF is used with WM in a Sankey diagram. (**B**) Target–drug interaction when QFPDT is used with WM in a Sankey diagram. (**C**) Target–drug interaction when NRICM101 is used with WM in a Sankey diagram.

Figure 2 shows 12 proteins on which CHM and WM affect simultaneously. The Sankey diagram (Figure 5) were created to investigate the possible drug–drug interaction between different kinds of medicine. All three CHM formulas had target proteins shared with Aspirin, Dexamethasone, and anticoagulant, and most were not COVID-related. Among these shared proteins, the majority of them were affected by antithrombotic drugs (Aspirin and Anticoagulant).

### 2.5. Proposed Molecular Pathways of the CHM and WM

**Figure 6:**
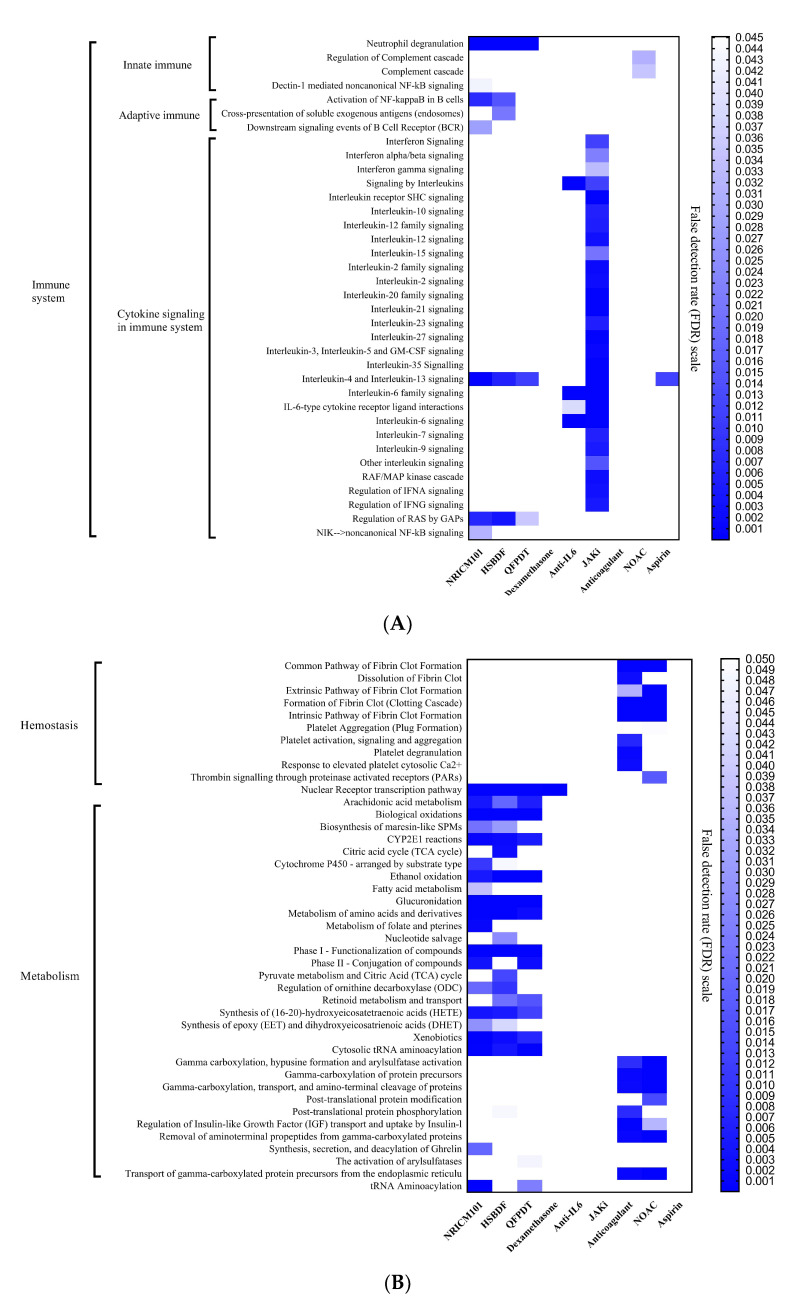
The differences in molecular pathways between CHM and WM. (**A**) The immune system-related molecular pathways. (**B**) The hemostasis and metabolism-related molecular pathways.

The potential molecular pathways of three CHM formulas and WM were proposed by considering binding proteins (Figure 6; for details, see Supplementary Material S6). For the immune system, CHM covered eight molecular pathways, while WM covered thirty. JAKi had a broad involvement in this area. Interestingly, the pathway of CHM and WM seemed quite different, and mutually complementary effects could be observed. WM mainly affected cytokine signaling-related pathways, while CHM mostly affected both innate and adaptive immunity-related pathways. Even in the innate immunity category, the complementary effects can still be found in which CHM affected neutrophil degranulation, and WM mainly affected complementary regulatory pathways. In hemostasis pathways, the CHM showed no impact on pathways associated with fibrin clot formation or platelet degranulation. For the pathways that affect metabolism, CHM and WM also showed different and mutually complementary effects.

**Figure 7:**
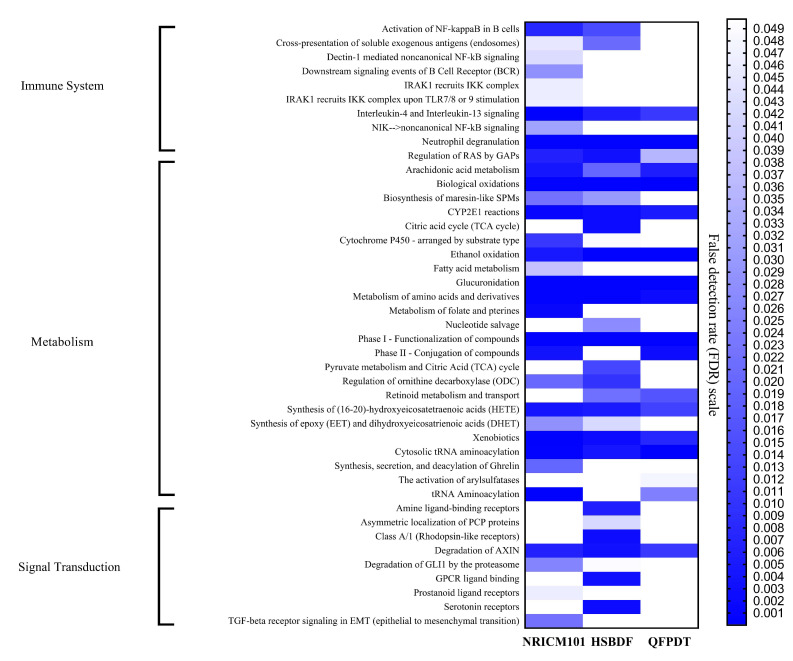
The molecular pathways covered by 3 CHM formulas.

To understand the shared and different mechanisms in the three CHM, Figure 7 compares the molecular pathways of these three drugs in the immune system as well as metabolism and signal transduction (for details, see Supplementary Material S7). NRICM101 covered the most pathways. QFPDT and NRICM101 shared similar pathways, and both of them showed mutually complementary effects with HSBDF in the signal transduction area. NRICM101 had a predominant role in the immune system, while HSBDF took a crucial part in signal transduction.

## 3. Discussion

By accentuating the disease-related proteins, the results showed the usefulness of CHM for COVID-19 and the feasibility of combining both WM and CHM when managing COVID-19. For all possible targets of both WM and CHM, CHM affected much more targets than WM, which may be relevant to a large number of ingredients in CHM formulas. A previous study reported that CHM had multi-target effects and, therefore, can meet the requirement to overcome diseases involving multi-system and complex pathways [26]. This corresponds with the holistic philosophy of CHM “bian zheng lun zhi” and “sovereign, minister, assistant and courier”, which means CHM prescriptions were made according to both the patient’s main discomfort and general conditions. Figure 2 summarizes the total target proteins covered by CHM and WM. CHM and WM mainly affected different proteins, and CHM may have a broader effect. Thirty-six COVID-related proteins are covered by WM or CHM, indicating possible pathways for existing drugs to fight COVID-19 and their therapeutic advantages. Sixty COVID-related proteins have no direct connection to the chosen drugs. This provides possible directions for future drug development.

As mentioned above, mutually complementary effects of CHM and WM in the immune system can be observed in our results. It is necessary to investigate how these drugs mediate the immune system. All three CHM formulas connected TLR3, TLR2, IL-6, and IL-8 (Figure 3). Toll-like receptors and neutrophils are crucial in innate immunity. One study mentioned that patients who had the mutation of gene TLR3 had more association with severe COVID [27]. Another study concluded that neutrophil-associated inflammation plays a vital role in Acute Respiratory Failure Syndrome (ARDS), and revealed that IL-6 and IL-8 take part in the mechanism of ARDS by upregulating neutrophil activation [28]. Neutrophil degranulation is strongly associated with 3 CHM formulas in Figure 6, implying that CHM has a potential benefit in ARDS compared to WM. TNF-α and IL-10 are two important proteins that both have four different kinds of CHM or WM (Figure 3) affect them respectively. A previous study suggested a reduction in T cell counts in COVID-19 patients and high levels of pro-inflammatory markers such as TNF-α, IL-6, IL-8, and IL-10 may be a potential reason for the killing of T cells [29]. Briefly, CHM and certain kinds of WM fight against COVID-19 through regulating T cell-mediated immune response, and CHM has an additional effect on innate immunity, especially by directly participating in neutrophil-involved pathways.

JAK is a group of kinases that plays an important role in the signal transduction of several immune pathways. In BMJ living review [1], it has been proven to have a benefit in decreasing mechanical ventilation and the duration of mechanical ventilation in COVID-19 patients. In our study (Figure 3), JAK1 and JAK2 were not linked to CHM, probably because there is no drug in the three chosen herbal formulas found to directly affect JAK in previous studies. After searching on other literatures, we found that some CHM extracts can act like JAKi, such as Notopterol, Forsythiaside A, Genkwanin, Tanshinone I, and Cinnamaldehyde [30,31,32,33,34,35]. These CHM may be potential candidates for new formulas to improve existing treatments.

One of the prominent manifestations of severe COVID-19 is the formation of thrombosis in vessels, leading to multiple organ failure. According to a previous study (36), the ACE/ACE2 ratio plays an important role in platelet aggregation and consequent thrombosis by causing vascular endothelium injury. The three CHM formulas have an impact on ACE (Figure 3), but have no direct influence on coagulation-related proteins such as CXCL7 and PLMN (Figure 1). Therefore, it is reasonable to expect that CHM can reduce thrombosis genesis without having the side effect of hemorrhage. This interpretation can also be verified by Figure 6 that CHM does not participate in any hemostasis pathways.

Obesity and metabolic syndrome have been proven to be related to poor outcomes in COVID-19 patients [36]. COVID-19 downregulates ACE2 expression and can eventually induce hyperglycemia [37]. Moreover, among the patients already diagnosed with diabetes mellitus (DM), better-controlled blood sugar was associated with significantly lower mortality than poorly controlled blood sugar [38]. All three CHM formulas not only affect ACE but also blood sugar control related target proteins such as DPP4 and GHSR (Figure 1 and Figure 3), meaning that they can help reduce the risk of precipitating hyperglycemia caused by COVID-19 as well as lower the blood sugar level of pre-existing DM. In addition, since Dexamethasone is a vital treatment to treat COVID-19 but produces an elevation of blood glucose concentration, extra advantages can be expected if CHM is prescribed concurrently.

As for COVID-19 survivors, they often face other problems. Long COVID, known as post-COVID-19 syndrome, means patients have fatigue, dyspnea, sleep difficulties, anxiety, and other organ complications that last for more than 3 months after COVID-19 onset, and is an important issue after the pandemic [39]. Long COVID is associated with higher levels of systemic pro-inflammatory markers like IL-6 [40]. One study also concluded that IL-6 has its potential to mediate the neuropsychiatric symptoms of long COVID [41]. In our research, the three CHM formulas all impact IL-6, which may reflect its character for the sequela.

There are 12 non-COVID-related proteins covered simultaneously by CHM and WM, which may give us a clue as to additional effects apart from treating COVID-19 infection itself. For example, a study pointed out that high levels of MMP9 and prothrombotic state are associated with pulmonary fibrosis [42,43], a sequela of COVID-19 which affect daily activities. The use of three CHM with Aspirin may be a feasible direction for treating pulmonary fibrosis. In order to validate the possible side effects of the drug–drug interaction, we searched the protein–side effect database ([44]; for details, see Supplementary Material S8). There is no sufficient evidence of severe side effects. CHM is usually associated with hepatic or renal damage, but no related protein is found in our report.

Some COVID-related proteins are not covered by CHM or WM (for details, see Supplementary Material S5), and these proteins can be potential targets in new drug development. In addition, there are still some drawbacks to this study. First, we only analyzed three representative CHM formulas from official guidelines in Taiwan and China with different treatment perspectives based on TCM theory. More CHM formulas can be searched to expand the results. Second, because research about COVID-19 is rising, more COVID-related protein data may be discovered, although we have stuck to the latest version of the online protein–ingredient interaction database. Third, monoclonal antibodies such as Casirivimab and Imdevimab were not included in the previous review according to eligibility criteria [1]. Anti-viral agents such as remdesivir, lopinavir, and ritonavir are not our focus because they attack the virus itself instead of regulating the physiological pathway of the human body.

## 4. Materials and Methods

### 4.1. Data Collection and Study Design

**Figure 8:**
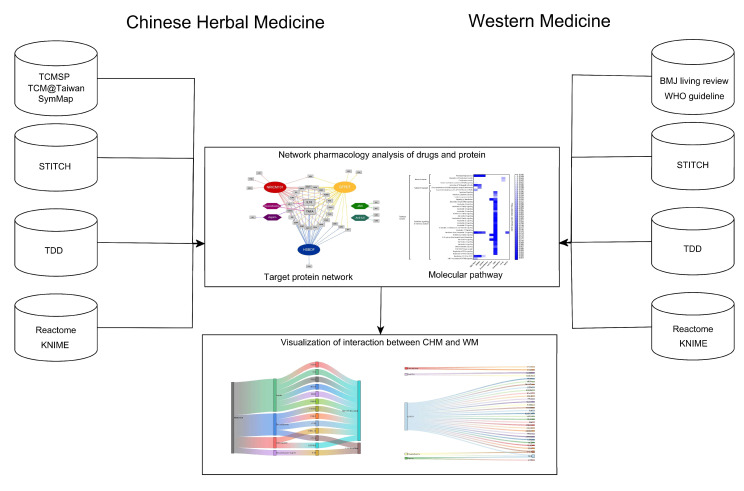
Flow diagram of this study.

A flow diagram of this study is shown in Figure 8. We manually searched works of literature on 3 representative CHM formulas: NRICM101, QFPDT, and HSBDF (last assessed date from PubMed: 30 April 2022). These 3 CHM formulas were chosen according to the Chinese Medicine Clinical Practice Guideline on COVID-19 in Taiwan [15] and the Diagnosis and Treatment Protocol for Novel Coronavirus Pneumonia (Trial Version 7) in China [14]. The ingredients of each CHM formula were acquired from published articles and TCMSP, SymMap, and TCM database@Taiwan [45,46,47]. On the other hand, 6 categories of WM were selected from BMJ living review ([1], last assessed on 6 April 2021) and WHO living guideline (last assessed on 30 September 2021). Dexamethasone, Anti-IL6 (Sarilumab and Tocilizumab), and JAKi (Baricitinib and Ruxolitinib) were selected as WM potentially effective for managing COVID-19 according to the clinical recommendations. Considering the common comorbidities of thrombosis events in COVID-19 patients, anticoagulant agents, including Heparin, Enoxaparin, and non-vitamin K antagonist oral anticoagulants (NOAC) as well as Aspirin were also included according to previous studies [48,49].

Furthermore, the Search Tool for Interacting Chemicals (STITCH) (25) was queried to acquire the possible target proteins of each ingredient from both WM and CHM. STITCH is a well-developed database composed of known and predicted connections between a chemical compound and target proteins derived from genomic context predictions, high-throughput lab experiments, gene co-expression databases, text mining in journal databases, and previous knowledge from other databases. STITCH contains 9.6 million proteins with interactions with over 430,000 chemical compounds, including ingredients of CHM. It inferred chemical–target protein connections from experiments with species other than humans, and it had a scoring system to describe the confidence of the connections. The scoring system, ranging from 0 to 1, summarizes the probability of connection occurrence by combining the probability from individual data sources, such as experiments on mice, text mining from journal databases, in a native Bayesian fashion. A higher score symbolizes stronger confidence in the connection between chemical compounds and target proteins. To pick the most confident connections between drug ingredients and the target proteins, a threshold of 0.800 is given [25].

In addition, to confirm the COVID-related proteins, we used the dataset released by the therapeutic database (TTD), containing 118 targets related to the illness associated with COVID and publicly available for drug selection for COVID [50]. These target proteins were used to classify WM/CHM-associated proteins as “COVID-covered” or “not related to COVID”.

### 4.2. Pharmacology Network Analysis

The connections between targets and CHM/WM were used to construct the networks. Cytoscape version 3.9.1 (NIGMS, Bethesda, MD, USA) was used to build and compare the network of CHM/WM and target proteins. First, we used all connections to construct an initial network pharmacology. This network would be used to compare the WM and CHM-centered clusters and to explore the similarities in the coverage of targets in WM and CHM. A Venn diagram was produced by BioVenn to illustrate the coverage of all targets between clusters of WM and CHM [51]. Second, COVID-related targets were used to construct networks in order to clarify the relationship between proteins and therapeutic agents. Furthermore, Sankey diagrams were created to illustrate drug interactions when 3 CHM formulas were prescribed with WM separately. Moreover, since CHM would be used with WM concurrently under most circumstances, and the WM-CHM interactions would become an issue when combining WM with either kind of CHM, the coverage of COVID-related targets of WM with QFBDT, NRICM101, and HSBDF were plotted by using a Sankey diagram as well.

### 4.3. Pathway Enrichment Analysis

To assess the overview molecular pathways for COVID-related targets of WM and CHM, the targets of each CHM and WM were used to perform the Gene Ontology (GO) and a pathway over-representation analysis by using the Reactome database [52,53,54]. The over-representation analysis is carried out on the hypothesis that if a molecular pathway is relevant, the proteins in the pathway should be more than randomly expected. The false discovery rate (FDR), calculated using the Benjamini–Hochberg approach, is used to demonstrate the statistical significance of each pathway. Pathways with FDR ≤ 0.05 would be consider significant. The differences between 3 CHM and WM in potential pathways and the comparisons of different pathways between 3 CHM formulas were performed separately. The freeware KNIME was used to manage the database and the application programming interface (API) was used to assess the latest information about the connections between CHM/WM and targets, including the pathway enrichment analysis [55].

## 5. Conclusions

Presently, this is the first study to explore the potential synergic effect of CHM and WM in COVID-19. In this study, we simulated the clinical situation by connecting network pharmacology to the biomedicine database and comparing the possible molecular pathways of CHM with WM on COVID-19. Considering the massive amount of ingredients and the variety of formulas in CHM, this result will provide a prediction model when treating a novel disease by CHM and WM.

## Figures and Tables

**Table 1:** Characteristics of the previous studies of the 3 CHM formulas and Western medicine.

Chinese medicine	Source	Ingredients	Clinical Studies	Sample Size (*n*)	Citation
NRICM101	Chinese Medicine Clinical Practice Guideline on COVID-19 in Taiwan	Ban Lan Gen, Yu Xing Cao, Huang Qin, Gua Lou, Jing Jie, Fang Feng, Sang Ye, Hou Po, Bo He, Gan Cao	Patients who had more risk factors and showing no improvement after 21 days of hospitalization achieved 3 consecutive negative results within a median of 9 days	33	Tsai KC et al. (2021)
QFPDT	Clinical Practice Guideline on COVID-19 in China	Ma Huang, Gan Cao, Ku Xing Ren, Shi Gao, Gui Zhi, Ze Xie, Zhu Ling, Bai Zhu, Fu Ling, Chai Hu, Huang Qin, Ban Xia, Sheng Jiang, Zi Wan, Kuan Dong Hua, She Gan, Xi Xin, Shan Yao, Zhi Shi, Chen Pi, Huo Xiang	↓50% COVID-19 related mortality ↓incidences of acute liver injury and acute kidney injury	8939	Yang RC et al. (2020)
HSBDF		Ma Huang, Ku Xing Ren, Shi Gao, Gan Cao, Huo Xiang, Hou Po, Cang Zhu, Cao Guo, Ban Xia, Fu Ling, Da Huang, Huang Qi, Ting Li Zi, Chi Shao	↓clinical remission time	40	Shi NN et al. (2021)
			↓median time of SARS-CoV-2 RNA clearance ↑ ratio of nucleic acid negative conversion ↓the high sensitivity C-reactive protein and serum ferritin	55	Wang Y et al. (2021)
Western medicine	Reference	Category	Effect		
Dexamethasone,	Western medicine from BMJ living review (until 6 APR 2021) and WHO guideline (until 24 September 2021)	Corticosteroid	↓mortality, ↓mechanical ventilation, ↑ventilator-free days		
Sarilumab, Tocilizumab		Anti-Interleukin-6 (Anti-IL6)	↓ mechanical ventilation, ↓duration of hospitalization		
Baricitinib, Ruxolitinib		Janus kinase inhibitor (JAKi)	↓ mechanical ventilation, ↓ duration of mechanical ventilation		
Aspirin	Hadid T et al. (2021) Meizlish ML et al. (2021)	Antiplatelet	prophylaxis of thrombosis		
UFH, LMWH (Enoxaparin)		Anticoagulant			
Rivaroxaban		Non-vitamin K antagonist oral anticoagulants (NOAC)			

NRICM101, National Research Institute of Chinese Medicine 101; QFPDT, Qing-Fei-Pai-Du-Tang; HSBDF, Hua-Shi-Bai-Du Formula. ↓ indicates an increase; ↑ indicates a decrease.

## Data Availability

Data is contained within the article and Supplementary Material.

## Supplementary Materials

The following supporting information can be downloaded at: https://www.mdpi.com/article/10.3390/ph15070794/s1, S1: Characteristics of included studies; S2: Composition-of-CHM-formula; S3: List-of-CHM-ingredients; S4: List-of-target-proteins; S5: List-of-target-proteins-not-covered-by-medicines; S6: Heatmap of CHM and WM; S7: Heatmap of CHM; S8: Side effect of drugs.
