# Supplementary figures and images for: The Potential Complementary Role of Using Chinese Herbal Medicine with Western Medicine in Treating COVID-19 Patients: Pharmacology Network Analysis
Source: Pharmaceuticals (Basel). 2022 Jun 26;15(7):794. doi: 10.3390/ph15070794 (PMC9323801; doi:10.3390/ph15070794)

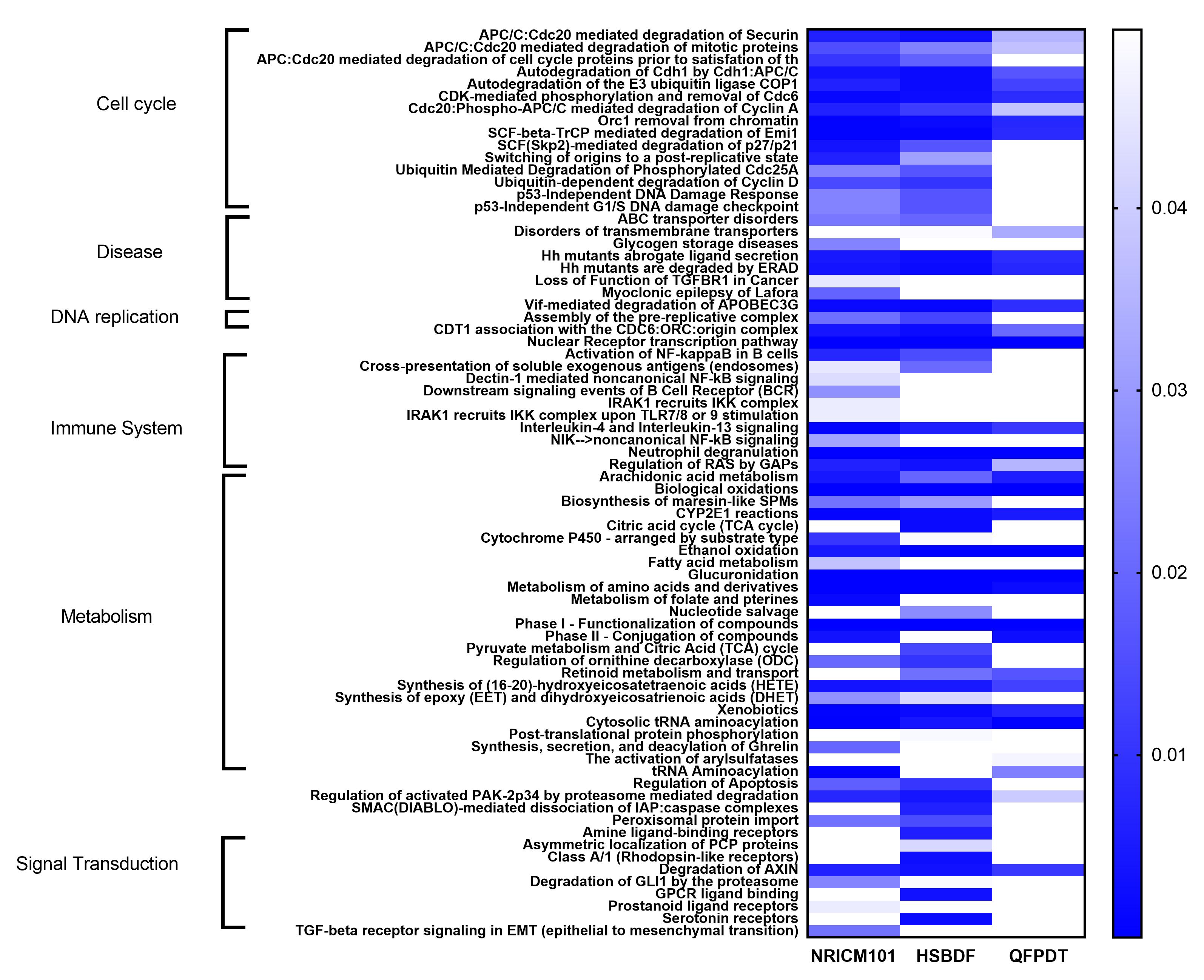

Supplement: Supplementary file 1 [file pharmaceuticals-15-00794-s001.zip › S7.heatmap of CHM.jpg]
